# Proliferating Pilar Tumor in an Atypical Location

**DOI:** 10.7759/cureus.114552

**Published:** 2026-08-14

**Authors:** Julio C Villarreal-Morin, Erick A Ramirez-Mendoza, Julio C Villarreal-Lozano

**Affiliations:** 1 Internal Medicine, ISSSTE Clínica Hospital Constitución, Monterrey, MEX; 2 Clinical Science, Universidad de Monterrey, San Pedro Garza García, MEX

**Keywords:** a case report, histo pathology, proliferating trichilemmal tumor, rare tumors, skin neoplasm

## Abstract

Proliferative pilar tumors are rare skin neoplasms with very few reported cases and, therefore, limited evidence. They derive from the outer root sheath of the hair follicle and usually manifest as a benign lesion although rarely they can become malignant. Macroscopically, they can be confused with a squamous cell carcinoma, being the histopathological assessment the main diagnostic tool to differentiate them. The aim of this case report is to present a case of malignant proliferative pilar tumor in an atypical location, as well as to describe the diagnostic and therapeutic approach. In this work, we highlight the importance of early histopathological diagnosis and timely surgical treatment.

## Introduction

Proliferating pillar tumors are exophytic tumors, mostly classified as solitary and benign lesions although there have been reported cases where they present as multiple lesions. The importance of its accurate diagnosis relies on the fact that they can be easily misdiagnosed as a squamous cell carcinoma. However, histopathologically, it differs by presenting characteristic trichilemmal keratinization. These tumors are found mainly in women over 40 years of age and their most frequent location is the scalp (up to 90%), so this case is an infrequent presentation. [1]. 

## Case presentation

**Figure 1:**
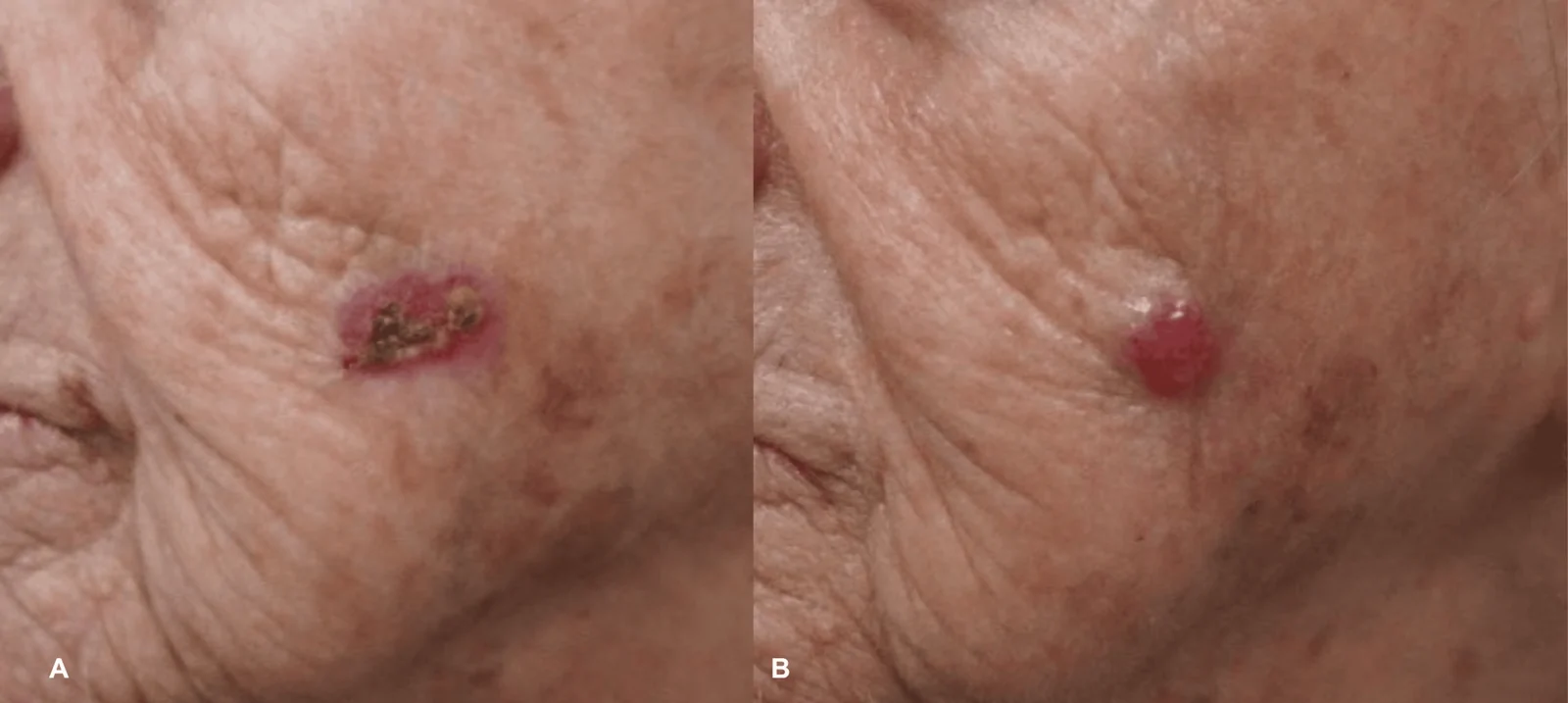
Dermatosis evolution (A) January 2025; (B) March 2025

**Figure 2:**
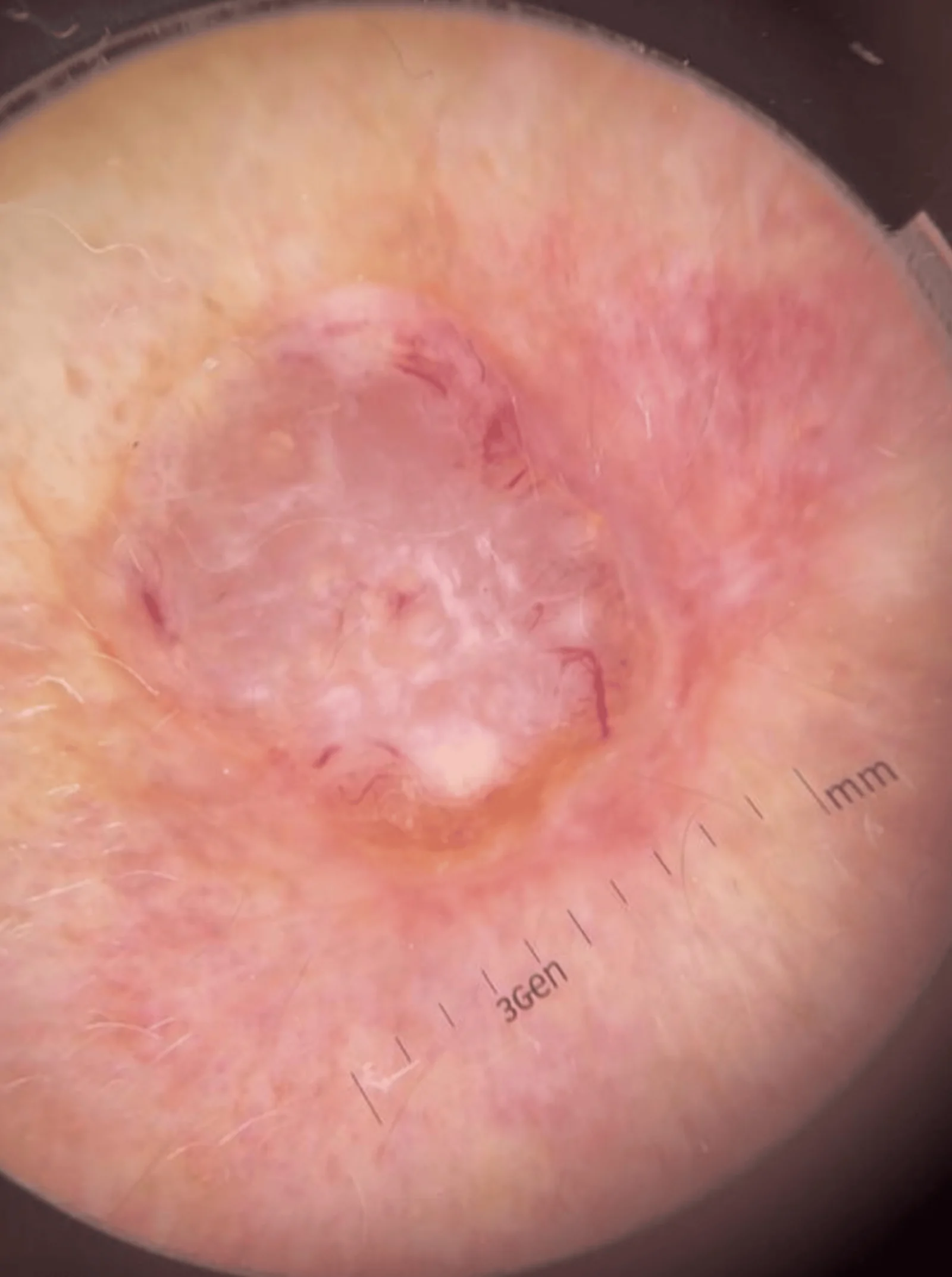
Dermoscopy

**Figure 3:**
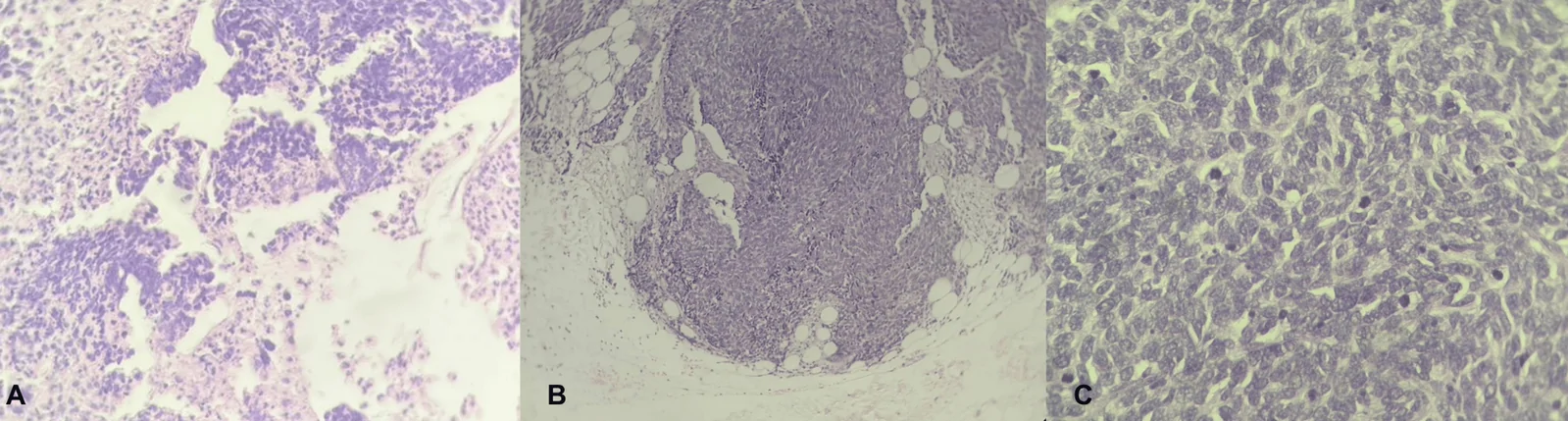
Photomicrography, H&E (A) 10x, central necrosis with absence of trichilemmal keratinization; (B) 10x, infiltration into adipose tissue; (C) 40x, tricholemic cells with slight pleomorphism H&E: Hematoxylin and eosin

We present the case of a 92-year-old woman with a past medical history of ischemic stroke (under current anticoagulant therapy), bilateral blindness secondary to glaucoma, diverticulosis and chronic sun damage. She presents to our office presenting a long-standing asymptomatic dermatosis located on her left cheek, characterized by an erythematous papule with central hyperkeratosis measuring 1.5 cm in diameter, which was first assessed in January 2025 (Figure 1). The patient was lost to medical follow-up, returning to our office two months later after presenting macroscopic changes, characterized by hyperplasia of the lesion (Figure 1). During questioning, she denied any trauma to the lesion. Dermoscopy with polarized light revealed a homogeneous nodular lesion with an erythematous base and a whitish-yellow central area, and the presence of arborescent blood vessels in its periphery (Figure 2). An excisional biopsy was performed, which revealed numerous atypical mitoses (15 to 20 per field), as well as tumor necrosis and hair follicles in the anagen phase (Figure 3). Based on the histological findings, a malignant proliferating pilar tumor was diagnosed with resection margins of 3.0 and 1.0 on the proximal laterals, 1.0 deep and 3.0 and 2.0 distal. After the first post-surgical visit (seven days later), the patient was lost to medical follow-up once again.

## Discussion

Proliferative pilar tumors are rare, benign exophytic tumors that can sporadically progress from a pre-existing pilar or tricholemmal cyst, originating in the outer root sheath of the hair follicle [1]. This is a rare, mostly benign neoplasm, with approximately 500 cases reported worldwide and a low potential for malignant transformation in 2% of cases. It was first described in 1966 by Wilson-Jones [2]. Epidemiologically, they occur most frequently in women between 50 and 75 years of age and are found primarily in patients over 60 years old. Clinically, they are solitary intradermal or subcutaneous lesions with a diameter of between 2 and 10 cm, though cases of multiple lesions have been reported. The main clinical differential diagnoses include epidermoid cyst, pilomatrixoma, keratoacanthoma, squamous cell carcinoma, and metatypical basal cell carcinoma, with histopathological evaluation being the main diagnostic tool. Histologically, a cyst with tricholemmal keratinization is observed, which is presented with compact amorphous keratin from the abrupt keratinization of the squamous cells that cover the wall of the cyst, which lacks a granular layer [3]. This type of keratinization is the main differentiating characteristic from squamous cell carcinoma. Based on their clinicopathological characteristics, primary papillary tumors (PPTs) are classified into three types according to their degree of behavior: benign, low-grade malignant, and high-grade malignant, the latter representing the type of tumor with the highest malignant biological behavior [2]. Benign lesions have little or no cellular atypia and no infiltration of the surrounding tissue. Low-grade malignant lesions form keratin globules that can compress adjacent cells as well as moderate cellular atypia. High-grade malignant lesions may present with marked atypia, nuclear pleomorphism, and lymphovascular infiltration. Often, these characteristics make it indistinguishable from squamous cell carcinoma; however, one of the main findings present in TPP at any grade is that histologically they preserve areas of tricholemal keratinization [4]. Immunohistochemistry represents another diagnostic tool, both to confirm malignancy and to differentiate it from squamous cell carcinoma [1]. This tumor shows positivity for the Ki-67 marker, indicating high cell proliferation, and low or no positivity for CD34, revealing poor cell differentiation [2] while the squamous cell carcinoma shows positivity for CK34β12/CK903, p40 and p63 [5].

Regarding treatment, wide excision of the lesion for subsequent histopathological identification of clear margins is the preferred therapeutic option. Given the high rate of local recurrence (20 to 30%), strict postoperative follow-up is essential [5]. One limitation present in our case was the loss of medical follow-up by the patient, which can be interpreted as a structural limitation, considering her age, comorbidities and, consequently, dependence due to her frail state. Another limitation we experienced was that immunohistochemical testing was not performed on the sample, which would have been ideal for diagnosis. The main objective of this study is to present a case of a rare cutaneous neoplasm with an atypical location and rapid progression to malignancy. The literature reports that, histologically, malignant pilar tumors, despite exhibiting atypia and cellular pleomorphism, continue to show areas of tricholemmal keratinization, which were absent in our patient's biopsy. This is because the tumor is ulcerated and, therefore, does not exhibit the characteristics of pillar differentiation. Due to the morphological similarity to squamous cell carcinoma, the importance of clinicopathological correlation for timely diagnosis and specific treatment is reinforced.

## Conclusions

In conclusion, diagnosing a proliferating pilar tumor remains challenging due to its similarity to other skin neoplasms, especially squamous cell carcinoma. Therefore, it should be considered in the differential diagnosis of exophytic lesions with progressive growth in older adults. The treatment of choice continues to be complete excision with clear margins. Clinical follow-up after the procedure is recommended due to the potential for recurrence. In this case, we wanted to highlight the atypical location of this tumor as well as the absence of tricheminal keratinization.
